# Public Perception on Artificial Intelligence–Driven Mental Health Interventions: Survey Research

**DOI:** 10.2196/64380

**Published:** 2024-11-28

**Authors:** Mahima Anna Varghese, Poonam Sharma, Maitreyee Patwardhan

**Affiliations:** 1 Department of Social Science and Language Vellore Institute of Technology Vellore India; 2 MEAL, Habitat for Humanity India Mumbai India

**Keywords:** public perception, artificial intelligence, AI, AI-driven, human-driven, mental health inteventions, mental health stigma, trust in AI, public perception, digital health, India, mobile phone

## Abstract

**Background:**

Artificial intelligence (AI) has become increasingly important in health care, generating both curiosity and concern. With a doctor-patient ratio of 1:834 in India, AI has the potential to alleviate a significant health care burden. Public perception plays a crucial role in shaping attitudes that can facilitate the adoption of new technologies. Similarly, the acceptance of AI-driven mental health interventions is crucial in determining their effectiveness and widespread adoption. Therefore, it is essential to study public perceptions and usage of existing AI-driven mental health interventions by exploring user experiences and opinions on their future applicability, particularly in comparison to traditional, human-based interventions.

**Objective:**

This study aims to explore the use, perception, and acceptance of AI-driven mental health interventions in comparison to traditional, human-based interventions.

**Methods:**

A total of 466 adult participants from India voluntarily completed a 30-item web-based survey on the use and perception of AI-based mental health interventions between November and December 2023.

**Results:**

Of the 466 respondents, only 163 (35%) had ever consulted a mental health professional. Additionally, 305 (65.5%) reported very low knowledge of AI-driven interventions. In terms of trust, 247 (53%) expressed a moderate level of Trust in AI-Driven Mental Health Interventions, while only 24 (5.2%) reported a high level of trust. By contrast, 114 (24.5%) reported high trust and 309 (66.3%) reported moderate Trust in Human-Based Mental Health Interventions; 242 (51.9%) participants reported a high level of stigma associated with using human-based interventions, compared with only 50 (10.7%) who expressed concerns about stigma related to AI-driven interventions. Additionally, 162 (34.8%) expressed a positive outlook toward the future use and social acceptance of AI-based interventions. The majority of respondents indicated that AI could be a useful option for providing general mental health tips and conducting initial assessments. The key benefits of AI highlighted by participants were accessibility, cost-effectiveness, 24/7 availability, and reduced stigma. Major concerns included data privacy, security, the lack of human touch, and the potential for misdiagnosis.

**Conclusions:**

There is a general lack of awareness about AI-driven mental health interventions. However, AI shows potential as a viable option for prevention, primary assessment, and ongoing mental health maintenance. Currently, people tend to trust traditional mental health practices more. Stigma remains a significant barrier to accessing traditional mental health services. Currently, the human touch remains an indispensable aspect of human-based mental health care, one that AI cannot replace. However, integrating AI with human mental health professionals is seen as a compelling model. AI is positively perceived in terms of accessibility, availability, and destigmatization. Knowledge and perceived trustworthiness are key factors influencing the acceptance and effectiveness of AI-driven mental health interventions.

## Introduction

### Background

Artificial intelligence (AI) is the most transformative tool of our times. It possesses the power to penetrate nearly every area imaginable. By leveraging the science and engineering of creating intelligent machines, AI has delivered promising results across various fields. From e-commerce and entertainment to navigation, health care, weather forecasting, agriculture, education, military, and marketing, AI has left no domain unexplored. From tracking steps on a digital watch to discovering personalized product recommendations on Amazon, AI has become an integral part of daily life [1]. In its various forms, deep learning has further revolutionized AI’s capabilities, introducing trained generative features that enable human-like interactions through chatbots across industries. According to Thormundsson [2], the AI market is projected to grow to over US $1.8 trillion by 2030. A report published in Forbes [3] highlighted that OpenAI’s ChatGPT has surpassed Netflix with 100 million active users. India ranks second globally with 6.32% of ChatGPT users, following the United States, which leads with 15.22%. The introduction of generative AI-powered teacher robots in India, such as “Iris” in Kerala and the Indus School robot in Karnataka, marks AI’s entry into education with a humanlike presence [4], captivating students’ imagination. Similarly, in the health sector, telehealth and telemedicine have gained significant traction worldwide, with many nations integrating them into their health care infrastructures. The COVID-19 pandemic accelerated the adoption of digital health apps, remote monitoring, and virtual consultations [5]. While some countries have made impressive strides in advancing AI applications in health care, others are still in the developmental stage.

### Health Care in India: Where We Are?

According to the Ministry of Health and Family Welfare [6], the doctor-to-patient ratio in India is approximately 1:834, based on the 80% availability of registered allopathic doctors and the inclusion of 5.65 lakh AYUSH (Ayurveda, Yoga, Naturopathy, Unani, Siddha, and Homeopathy) practitioners. India, one of the most diverse countries in the world, faces a significant health care challenge due to its large and growing population.

On average, countries allocate about 2% of their total budget to health care annually [7]. India is undeniably home to some of the world’s best doctors and has a health care system comprising public, private, nongovernmental organizations, charity, and public-private partnership models. However, meeting the diverse and growing needs of its vast population remains a significant challenge. Similar to other nations, India faces pressing health concerns such as diabetes, hypertension, and cancer. There is a noticeable shift from communicable to noncommunicable diseases in India. The country is striving to extend health care to underserved populations through government initiatives such as Ayushman Bharat Yojana, Aam Aadmi Bima Yojana, Rashtriya Kishor Swasthya Karyakram, and Mission Indradhanush [8]. However, as highlighted in an article published in Economic and Political Weekly [9], significant disparities persist between rural and urban areas, across genders, and in reaching marginalized communities. To address the nation’s health care needs, innovative, cost-effective, and socially accepted approaches are essential.

### AI in Health Care

AI in health care has the potential to significantly enhance the accuracy and efficiency of disease diagnosis and treatment. According to a critical review by Mirbabaie et al [10], AI applications can improve diagnostic precision and streamline processes. Currently, AI is widely used in health care for medical imaging analysis, drug discovery, robotic surgeries, assisting health care professionals, patient monitoring, and health assistant chatbots. Since the groundbreaking cardiac surgery involving a robotic arm in 1998 in India, the use of robots in medical procedures has gained significant popularity, particularly in surgery. Alongside robotics, AI is playing a transformative role in India’s health care revolution. Hospitals such as Manipal are leveraging IBM Watson’s extensive medical knowledge and data analytics capabilities, paving the way for future medical innovations in the country. With an estimated compound annual growth rate of 20% and rising demand for automation, the surgical robotics industry is projected to grow 5-fold by 2025, signaling a promising future for AI-driven medical advancements in India [11].

### Mental Health Burden and Countries’ Response

In a nation like India, with a population of 1.4 billion, public health concerns have been mounting due to the steady rise in mental health issues in recent years [12]. The COVID-19 pandemic in 2020 led to a sharp increase in cases of anxiety and depression. According to early forecasts by the World Health Organization, anxiety disorders were projected to rise by 26%, while severe depressive disorders were expected to increase by 28% within a year. Estimates suggest that around 15% of Indians have a mental health condition [12]. According to the National Mental Health Survey conducted by the National Institute of Mental Health and Neurosciences (NIMHANS) in 2015-16 [13], approximately 197.3 million Indians were affected by mental illnesses. Among them, 45.7 million experienced depressive disorders and 44.9 million had anxiety disorders [14]. An estimated 150 million people require mental health services, but less than 30 million are currently seeking care. Since 1990, the proportion of individuals impacted by mental health conditions has nearly doubled [15].

Despite significant efforts, the ratio of psychiatrists to the population continues to worsen each day. In response to this urgent demand, the Indian government allocates only 0.06% of its total health care budget to mental health. There are just 0.75 psychiatrists for every 100,000 citizens, with even fewer psychologists, social workers, and nurses specializing in mental health [16]. Developing human resources in the field of mental health presents its own set of costs and challenges. Research conducted in 2023 on 787 medical students from North India revealed that 37.2% had considered suicide, 10.9% had intentions to do so, and 3.3% had actually attempted it [16]. The increase in hidden mental health issues, such as suicidal ideation; aggression; and the use of tobacco, alcohol, and other drugs, underscores the urgent need to prioritize and adapt approaches to promote and provide adequate mental health services [17].

The growing demand for mental health care has opened up opportunities for the development of digital technologies and data-driven innovations to complement traditional in-person and telehealth services in psychology and psychiatry [13]. In line with this digital revolution, the Indian central government announced the National Tele-Mental Health Program (NTMHP) in its Union Budget for 2022-2023. In collaboration with the NIMHANS and the International Institute of Information Technology (IIIT), Bangalore, the program aims to enhance access to high-quality mental health counseling and care services [18].

### Barriers to Seeking Mental Health Support

Addressing mental health concerns in India is complicated by various social and cultural factors, including stigma, prejudice, gender disparity, poverty, rapid urbanization, and cultural perceptions of mental illness. Stigma, in particular, can discourage individuals from seeking treatment by causing them to distrust medical professionals [19]. Access to mental health services is further hindered by significant disparities in the availability and quality of mental health care, as well as the shortage of mental health professionals, especially in rural areas [12,20]. AI in the mental health industry holds the potential to address several challenges related to the availability, appeal, and accessibility of mental health treatments. However, there are still many unanswered questions about how to effectively apply and leverage AI to improve mental health care services, providers, and outcomes for clients [21].

### Artificial Intelligence in Mental Health Care

Currently, AI-driven mental health interventions are most commonly seen in the form of mobile-based mental health apps. Given the proven efficacy of AI in the health care field, applying the same technology to mental health care could offer significant benefits for humanity.

Beginning in the 1960s, ELIZA, a computer program, was developed to simulate a psychotherapist’s conversational skills. In 1971, another computer model was created to mimic paranoia during a diagnostic psychiatric interview, aiming to describe paranoid behavior [22]. Today, gaming modalities target cognitive and psychosocial domains, focusing on specific weaknesses in various psychiatric conditions, including biofeedback, behavioral modification, social motivation, cognitive behavioral therapy, and attention improvement [23]. Applications such as MindLAMP and BiAffect are used for assessment and recovery, and they also predict manic and depressive episodes in patients with bipolar disorder [24].

By contrast, chatbots are another type of AI technology that has gained popularity in providing greater access to treatment for psychiatric conditions. Examples are Woebot, Tess, new avatar therapies, Replika, the robotic seal Paro, and eBear, which use texting to guide individuals through difficult emotional moments [22]. In India, several digital start-ups, such as Inner Hour, Trijog, “IWill,” YourDOST, Wysa, and TalktoAngel, have been developed to address mental health issues.

### Public Perception as a Key Component in AI-Driven Mental Health Interventions

Public perception plays a crucial role in shaping attitudes that can either facilitate or hinder the use of new technology. According to Cheung and Vogel [25], a positive perspective from users increases their intention to use technology for learning. Attitude is a key factor in technology usage [26,27]. Similarly, public perception and acceptance of AI-driven mental health care are essential in determining the effectiveness and adoption of such technologies. Public perception and attitudes toward AI in medical care can influence the early development of AI products [28]. Recent studies suggest a positive correlation between the widespread use of AI technologies in the medical field and the favorable perceptions and acceptance of AI among users [29].

Research indicates that racial and ethnic minority groups are more concerned about potential misdiagnoses, privacy breaches, fewer doctor-patient visits, and higher costs—even though the general public supports AI’s potential to improve health care [30,31]. The ethical and social considerations surrounding the use of AI and robotics in mental health care are complex and multifaceted. Fiske et al [31] described AI as a viable method for treating mental health disorders in recent research, but they also highlighted several ethical and societal concerns related to its application (also see [32]).

The use of AI in mental health care has the potential to enhance decision-making, tailor personalized treatments, and optimize user experiences in web and smartphone services for mental health care [33]. In a study conducted in the United Kingdom among therapists, it was reassuring to see that, despite their criticisms of these technological advancements, the majority of participants were open to the potential benefits AI and computers may offer in the future [34].

While research on AI is increasing, only a small number of studies specifically address how the general population views AI, particularly in the field of mental health care [34]. A better understanding of public perception within the Indian population could guide the implementation of AI-driven mental health interventions in India. In light of these gaps in the literature [30-34], and considering that this is a new field with significant potential for future growth, current exploration is urgently needed. This study is formative research on public perceptions of and attitudes toward existing AI-driven mental health interventions. It explores opinions on the use of AI-driven mental health services and compares perceptions of their future applicability with traditional, human-based mental health interventions in India.

### Study Objectives

This study aims to (1) analyze public perceptions of AI-driven mental health interventions; (2) compare public opinions on human-based mental health interventions and AI-based mental health interventions; (3) examine the relationships among various factors that influence perceptions of human-based and AI-driven mental health interventions; and (4) qualitatively explore opinions on the benefits and limitations of AI-driven mental health interventions.

## Methods

### Study Population and Recruitment

A total of 466 participants (269 females and 197 males) were selected for this study using an online convenience, nonprobability sampling method. Participants who were unfamiliar with the concept of AI were excluded in the initial stages, as the study does not rely on random selection from a well-defined population [35]. Social media surveys have become increasingly popular for gathering opinions and observational data during and after the COVID-19 pandemic. Given the nature of our research, it was essential to target individuals who are familiar with the concept of AI in mental health, a relatively new area. As AI in mental health is not yet widely known, traditional methods of data collection would have made it challenging to recruit participants from the target population. Therefore, an online web survey, shared via social and professional online platforms, allowed us to more effectively reach and engage the ideal population. The Step-by-Step Guide of the American Psychological Association by Kühne and Zindel [35] was consulted before the survey was launched. Studies confirm that nonprobability sampling not only reduces costs but is also effective in recruiting survey participants [36]. Research has demonstrated higher response rates in online surveys compared with traditional methods, owing to the ease of access, convenience, and availability of online forms. The “LGBielefeld” survey project [36,37], targeting the LGBTQ (lesbian, gay, bisexual, transgender, and queer or questioning) population, exemplifies the success of online surveys in reaching and recruiting hard-to-reach participants through social media.

Eligibility for participation in the study was restricted to adult residents of India who provided informed consent and had an understanding of the concept of AI in mental health. Participants were categorized into various age groups. Responses to the survey were received from participants across India, including urban (n=308), semiurban (n=92), and rural (n=66) areas.

Recruitment strategies involved the distribution of informational flyers through online platforms. These flyers, available in English, were shared on social media sites such as Reddit (Reddit, Inc.), Instagram (Meta Platforms, Inc.), and LinkedIn (Microsoft Corp.). Additionally, word-of-mouth referrals from initial research participants helped recruit further volunteers.

### Measures

#### Demographic Details

The demographic data collected from participants included their initials, gender, age (years), education level, field of study, place of residence, and familiarity with the term “AI.”

#### Survey

The survey was developed following a pilot study and expert validation. It comprised 2 open-ended questions and 28 close-ended questions, focusing on the following domains: Knowledge and Awareness About AI-Driven Mental Health Interventions, Trust in AI-Driven Mental Health Interventions, Trust in Human-Based Mental Health Interventions, Effectiveness of AI in Mental Health Management, Stigma Associated With AI-Driven Mental Health Interventions, Stigma Associated With Human-Based Mental Health Services, Societal Acceptance and Likelihood of Future Use of AI, and Purpose of Using AI-Driven Mental Health Interventions. Of the 30 questions, 2 were open-ended, asking participants about the benefits and concerns associated with using AI-driven mental health interventions. The remaining questions utilized a 5-point Likert scale. All domains were interpreted based on low, moderate, and high scores. Reverse scoring was applied to the domains of Trust in AI-Driven Mental Health Interventions, Trust in Human-Based Mental Health Interventions, Stigma Associated With AI-Driven Mental Health Interventions, and Stigma Associated With Human-Based Mental Health Services. High scores indicated a more positive perception of the respective domain.

### Data Collection

Participants who agreed to participate in the study were asked to complete the Google Form (Google LLC/Alphabet Inc.), which was sent through the researcher’s email account. Google Forms proved to be an excellent option, as it allowed for the seamless analysis of the survey data in real-time, without the need for manual tabulation or coding [37]. Data were collected from participants between November and December 2023. In the electronic survey, participants responded to questions regarding their perceptions, as well as demography-related questions. All data collection forms were regularly reviewed by the primary investigator (MAV and PS) to ensure their credibility, completeness, and to eliminate any duplication.

### Statistical Analysis

#### Quantitative Analysis

Data analysis was performed using SPSS Software for Windows (IBM Corp.). Descriptive statistics, including both univariate and bivariate analyses, were used to interpret the data. Univariate analysis was conducted to examine participant demographics, as well as gender-wise and location-wise distributions across various domains. Bivariate analysis was used to explore relationships between different domains, and Pearson correlation analysis was used to assess the correlations between various variables.

#### Qualitative Analysis

Qualitative analysis was conducted to assess and categorize the public’s opinions regarding the purpose of using AI-driven mental health interventions, as well as the benefits and concerns associated with these interventions.

### Ethical Consideration

The study adhered to the Helsinki Declaration (1964) Code of Ethics. It was a general opinion survey conducted among consenting adults aged 18 and above, posing minimal to no risk to participants. As such, an ethical exemption was granted by the Institutional Review Board (IECH No: VIT/IECH/2024/16 IECH/24 September 2024). Online informed consent was obtained from all participants involved in the study via the researcher’s account. All data collected, including participants’ initials, were kept confidential and accessible only to the research team.

## Results

### Overview

The results section is divided into 4 parts. The first section presents the univariate analysis of the data, including participant demographics and a breakdown of knowledge and perceptions across different categories, such as location and gender. In the second section, bivariate analysis is used to explore the associations between different domains and gender, as well as location. The third section applies Pearson correlation analysis to examine the relationships between variables. Lastly, the fourth section discusses participants’ opinions on the benefits, concerns, and future uses of AI-driven mental health interventions.

### Univariate Analysis Results: Participant Demographics

Table 1 presents the responses from 467 Indian participants. The majority of participants were female (269/467, 57.6%). The transgender participant was excluded from the analysis due to the lack of representativeness. The largest group of participants fell within the age range of 16-25 years (218/466, 46.8%). Age categories were determined based on the median, as the population using AI was concentrated around this age range. A study by the Reuters Institute and Oxford University [38] found that AI usage was most prevalent among individuals aged 18-24 years, supporting the rationale for selecting this age range. The majority of responses came from urban areas (308/466, 66.1). Postgraduates represented the largest group (254/466, 54.5%). The inclusion criteria required participants to be familiar with the concept of AI.

**Table 1:** Participants’ demographics and prior experience with a mental health professional.

Variable			Values, n (%)
**Gender (N=467)**			
	Female	269 (57.6)	
	Male	197 (42.2)	
	Transgender	1 (0.2)	
**Age (years; N=466)**			
	16-25	218 (46.8)	
	26-35	92 (19.7)	
	36-45	87 (18.7)	
	46-55	49 (10.5)	
	56-65	20 (4.3)	
**Education (N=466)**			
	High school	14 (3.0)	
	Undergraduate	130 (27.9)	
	Postgraduate	254 (54.5)	
	Doctorate	68 (14.6)	
**Location (N=466)**			
	Rural	66 (14.2)	
	Semiurban	92 (19.7)	
	Urban	308 (66.1)	
**Consulted mental health professional (N=466)**			
	Yes	163 (35.0)	
	No	303 (65.0)	

### Descriptive Statistics

Tables 2 and 3 present the gender-wise and location-wise analysis, respectively, of public knowledge and perceptions across various domains. It was found that 305 of 466 (65.5%) participants had low Knowledge and Awareness About AI-Driven Mental Health Interventions. Regarding Trust in AI-Driven Mental Health Interventions, of the 466 participants, 247 (53%) exhibited a moderate level of trust, while 309 (66.3%) expressed a moderate level of Trust in Human-Based Mental Health Interventions. As many as 304 (65.2%) participants believed that AI-driven interventions would be moderately effective. Regarding stigma, 392 (84.1%) reported a moderate level of stigma, while 242 (51.9%) indicated a high level of stigma toward human-based interventions (low scores indicate high stigma). Additionally, 304 (65.2%) felt that social acceptance and the likelihood of using AI-driven mental health interventions would be low.

**Table 2:** Perception toward various domains of AI^a^-driven mental health interventions (gender-wise analysis; N=466)^b,c^.

Levels		Domains						
		Knowledge and Awareness	Trust in AI-Driven Interventions	Trust in Human-Based Mental Health Interventions	Effectiveness of AI in Mental Health Management	Stigma While Using AI-Driven Interventions	Stigma While Using Human-Based Interventions	Societal Acceptance and Likelihood of Future Use of AI and Use of AI
**Low, n (%)**								
	Males	138 (70.1)	70 (35.5)	17 (8.6)	51 (25.9)	8 (4.1)	99 (50.3)	134 (68.0)
	Females	167 (62.1)	125 (46.5)	26 (9.7)	59 (21.9)	16 (5.9)	143 (53.2)	170 (63.2)
	Total	305 (65.5)	195 (41.8)	43 (9.2)	110 (23.6)	24 (5.2)	242 (51.9)	304 (65.2)
**Moderate, n (%)**								
	Males	47 (23.9)	113 (57.4)	140 (71.1)	122 (61.9)	168 (85.3)	72 (36.5)	44 (22.3)
	Females	78 (29.0)	134 (49.8)	169 (62.8)	182 (67.7)	224 (83.3)	90 (33.5)	72 (26.8)
	Total	125 (26.8)	247 (53.0)	309 (66.3)	304 (65.2)	392 (84.1)	162 (34.8)	116 (24.9)
**High, n (%)**								
	Males	12 (6.1)	14 (7.1)	40 (20.3)	24 (12.2)	21 (10.7)	26 (13.2)	19 (9.6)
	Females	24 (8.9)	10 (3.7)	74 (27.5)	28 (10.4)	29 (10.8)	36 (13.4)	27 (10)
	Total	36 (7.7)	24 (5.2)	114 (24.5)	52 (11.2)	50 (10.7)	62 (13.3)	46 (9.9)

^a^AI: artificial intelligence.

^b^Number of males=197.

^c^Number of females=269.

**Table 3:** Perception toward various domains of AI^a^-driven mental health interventions (location-wise analysis; N=466)^b,c,d^.

Domains		Knowledge and Awareness	Trust in AI-Driven Mental Health Interventions	Trust in Human-Based Interventions	Effectiveness of AI in Mental Health Management	Stigma While Using AI-Driven Interventions	Stigma While Using Human-Based Interventions	Societal Acceptance and Likelihood of Future Use of AI
**Low n (%)**								
	Rural	41 (62.1)	21 (31.8)	3 (4.5)	15 (22.7)	2 (3.0)	32 (48.5)	39 (59.1)
	Semi- urban	62 (67.4)	40 (43.5)	9 (9.8)	26 (28.3)	2 (2.2)	47 (51.1)	69 (75)
	Urban	202 (65.6)	134 (43.5)	31 (10.1)	69 (22.4)	20 (6.5)	163 (52.9)	196 (63.6)
	Total	305 (65.5)	195 (41.8)	43 (9.2)	110 (23.6)	24 (5.2)	242 (51.9)	304 (65.2)
**Moderate n (%)**								
	Rural	19 (28.8)	43 (65.2)	54 (81.8)	44 (66.7)	58 (87.9)	22 (33.3)	18 (27.3)
	Semi- urban	25 (27.2)	46 (50.0)	61 (66.3)	53 (57.6)	77 (83.7)	32 (34.8)	13 (14.1)
	Urban	81 (26.3)	158 (51.3)	194 (63.0)	207 (67.2)	257 (83.4)	108 (35.1)	85 (27.6)
	Total	125 (26.8)	247 (53.0)	309 (66.3)	304 (65.2)	392 (84.1)	162 (34.8)	116 (24.9)
**High n (%)**								
	Rural	6 (9.1)	2 (3.0)	9 (13.6)	7 (10.6)	6 (9.1)	12 (18.2)	9 (13.6)
	Semi- urban	5 (5.4)	6 (6.5)	22 (23.9)	13 (14.1)	13 (14.1)	13 (14.1)	10 (10.9)
	Urban	25 (8.1)	16 (5.2)	83 (26.9)	32 (10.4)	31 (10.1)	37 (12.0)	27 (8.8)
	Total	36 (7.7)	24 (5.2)	114 (24.5)	52 (11.2)	50 (10.7)	62 (13.3)	46 (9.9)

^a^AI: artificial intelligence.

^b^Number of rural people=66.

^c^Number of semiurban people=92.

^d^Number of urban people=308.

### Bivariate Analysis: Chi-Square Test for Significance

Knowledge and Awareness About AI-Driven Mental health Interventions

A chi-square test of independence was performed to assess the relationship between Knowledge and Awareness About AI-Driven Mental Health Interventions and age, as shown in Table 4. A significant relationship was found between the 2 variables: *χ*^2^ (N=466)=6.2 (*P*=.04). When the chi-square test of independence was conducted between the Knowledge and Awareness About AI-Driven Mental Health Interventions domain and gender, no significant relationship was observed: *χ*^2^ (N=466)=3.4 (*P*=.18). However, a higher percentage of males (138/197, 70.1%) had low knowledge and awareness about AI-driven mental health interventions compared with females (167/269, 62.1%), as shown in Table 2. Table 3 shows the frequency and percentages of data between location and the Knowledge and Awareness About AI-Driven Mental Health Interventions domain. It was observed that the data were skewed toward urban areas, but it was also noted that individuals from rural (41/66, 62%), urban (202/308, 65.6%), and semiurban (62/92, 67%) areas all had low levels of awareness.

**Table 4:** Chi-square test between age and Knowledge and Awareness About Artificial Intelligence–Driven Mental Health Interventions domain (valid cases=466).

Test	Value (*df*)	Significance (2-sided)
Pearson chi-square	6.2 (2)	.04
Likelihood ratio	6.251 (2)	.04
Linear-by-linear association	5.267 (1)	.02

Trust in AI-Driven Mental Health Interventions

From the chi-square test of independence performed to assess the relationship between Trust in AI-Driven Mental Health Interventions and age, it was observed that there was no significant relationship between the 2 variables: *χ*^2^ (N=466)=4.7 (*P*=.09). Table 5 shows that when conducting the chi-square test of independence between Trust in AI-Driven Mental Health Interventions and gender, a significant relationship was found between these variables: *χ*^2^ (N=466)=7.0 (*P*=.03). Table 3 shows the cross-tabulation between location and Trust in AI-Driven Mental Health Interventions. Although the data are skewed toward the urban population, it was observed that individuals from all 3 locations exhibited a moderate level of Trust in AI-Driven Mental Health Interventions.

**Table 5:** Chi-square test between gender and the Trust in Artificial Intelligence–Driven Mental Health Interventions domain (number of valid cases=466).

Test	Value (*df*)	Significance (2-sided)
Pearson chi-square	7.008 (2)	0.03
Likelihood ratio	7.014 (2)	0.03
Linear-by-linear association	6.944 (1)	0.008

Trust in Human-Based Mental Health Interventions

The results of the chi-square test between age and the Trust in Human-Based Mental Health Interventions domain showed no significant relationship between the 2 variables: *χ*^2^ (N=466)=2.8 (*P*=.24). The chi-square test between gender and Trust in Human-Based Mental Health Interventions showed no significant relationship: *χ*^2^ (N=466)=3.7 (*P*=.16). However, as observed in Table 2, both males (140/197, 71.1%) and females (169/269, 62.8%) exhibited a moderate level of Trust in Human-Based Mental Health Interventions. Table 3 presents the frequency of individuals who have Trust in Human-Based Mental Health Interventions across rural, urban, and semiurban areas. It was observed that people in rural (54/66, 82%), urban (194/308, 63%), and semiurban (61/92, 66%) areas displayed a moderate level of trust in these interventions.

Effectiveness of AI in Mental Health Management

Table 6 presents the results of the chi-square test between age and the Effectiveness of AI in Mental Health Management. It shows a significant relationship between age and the perceived Effectiveness of AI in Mental Health Management: *χ*^2^ (N=466)=10.5 (*P*=.005). By contrast, the chi-square test between gender and the Effectiveness of AI in Mental Health Management domain did not show a significant relationship: *χ*^2^ (N=466)=1.6 (*P*=.40). However, as shown in Table 2, both males (122/197, 61.9%) and females (182/269, 67.7%) perceived AI-driven interventions to have a moderate level of effectiveness in mental health management. Table 3 presents the distribution of opinions on the Effectiveness of AI in Mental Health Management across rural, urban, and semiurban areas. It was observed that individuals from all locations believed AI-driven mental health interventions had a moderate level of effectiveness.

**Table 6:** Chi-square test between age and Effectiveness of Artificial Intelligence in Mental Health Management (number of valid cases=466).

Test	Value (*df*)	Significance (2-sided)
Pearson chi-square	10.597 (2)	.005
Likelihood ratio	10.757 (2)	.005
Linear-by-linear association	10.061 (1)	.002

Stigma Associated With AI-Driven Mental Health Interventions

The chi-square test between age and Stigma Associated With AI-Driven Mental Health Interventions revealed no significant relationship between these variables: *χ*^2^ (N=466)=2.7 (*P*=.26). Similarly, the chi-square tests showed no significant relationship between gender and Stigma Associated With AI-Driven Mental Health Interventions: *χ*^2^ (N=466)=0.8 (*P*=.65). It was observed in Table 2 that both males (168/197, 85.3%) and females (224/269, 83.3%) exhibited a moderate level of stigma when using AI-driven mental health services. Table 3 presents the frequency and percentage of people from rural, semiurban, and urban areas who also displayed a moderate level of stigma toward AI-driven mental health services. Regardless of location, the data show that individuals from rural (58/66, 88%), semiurban (77/92, 84%), and urban (257/308, 83.4%) areas demonstrated a similar level of stigma.

Stigma Associated With Human-Based Mental Health Interventions

Table 7 presents the chi-square test results between age and Stigma Associated With Human-Based Mental Health Interventions. A significant relationship was found between age and Stigma Associated With Human-Based Mental Health Interventions: *χ*^2^ (N=466)=10.7 (*P*=.005). However, the chi-square test between gender and Stigma Associated With Human-Based Mental Health Interventions revealed no significant difference between the variables: *χ*^2^ (N=466)=0.5 (*P*=.78). Although both males (99/197, 50.3%) and females (143/269, 53.2%) expressed a high level of stigma toward human-based mental health interventions, as shown in Table 2, Table 3 illustrates the cross-tabulation between location and stigma toward human-based mental health services. It was observed that people from rural (32/66, 48%), urban (163/308, 52.9%), and semiurban (47/92, 51%) areas all reported experiencing high stigma toward human-based mental health services.

**Table 7:** Chi-square test between age and Stigma Associated With Human-Based Mental Health Interventions (number of valid cases=466).

Test	Value (*df*)	Significance (2-sided)
Pearson chi-square	10.777 (2)	.005
Likelihood ratio	10.830 (2)	.004
Linear-by-linear association	2.979 (1)	.084

Societal Acceptance and Likelihood of Future Use of AI

The chi-square test between age and societal acceptance, as well as the likelihood of future use of AI, revealed no significant relationship between age and these factors: *χ*^2^ (N=466)=2.6 (*P*=.27). Similarly, the chi-square test between gender and societal acceptance, as well as the likelihood of future use of AI, showed no significant relationship between these variables: *χ*^2^ (N=466)=1.3 (*P*=.52). It was observed that both males (134/197, 68%) and females (170/269, 63.2%) exhibited low societal acceptance and likelihood of future use of AI, as shown in Table 2. Table 3 presents the cross-tabulation between location and societal acceptance, as well as the likelihood of future use of AI. It was found that individuals from rural (39/66, 59%), urban (196/308, 63.6%), and semiurban (69/92, 75%) areas similarly reported low societal acceptance and likelihood of future use of AI.

### Pearson Correlation Analysis

Table 8 shows the correlation between various variables. A significant moderate positive correlation was observed between Knowledge and Awareness About AI-Driven Mental Health Interventions and Trust in AI-Driven Mental Health Interventions, *r*=0.35 (N=466; *P*<.001), as well as between Knowledge and Awareness About AI-Driven Mental Health Interventions and acceptance of AI, *r*=0.39 (N=466; *P*<.001). Additionally, a significant but very weakly positive relationship was found between Knowledge and Awareness About AI-Driven Mental Health Interventions and the perceived Effectiveness of AI in Mental Health Management, *r*=0.19 (N=466; *P*<.001). Considering the variables Trust in AI-Driven Mental Health Interventions and Trust in Human-Based Mental Health Interventions, a significant but very weak negative relationship was observed between the 2 variables, *r*=–0.13 (N=466; *P*=.005). By contrast, a strong positive relationship was found between Trust in AI-Driven Mental Health Interventions and the perceived effectiveness of these interventions, *r*=0.57 (N=466; *P*<.001). Additionally, there was a significant moderate positive relationship between Trust in AI-Driven Mental Health Interventions and acceptance of AI-driven mental health interventions, *r*=0.47 (N=466; *P*<.001). It was also observed that there is a significant but very weak negative relationship between Trust in Human-Based Mental Health Interventions and the Effectiveness of AI in Mental Health Management, *r*=–0.15 (N=466; *P*=.001). Additionally, a weakly negative relationship was found between Trust in Human-Based Mental Health Interventions and stigma associated with these AI-driven interventions, *r*=–0.13 (N=466; *P*=.006). A similarly weak negative relationship was observed between Trust in Human-Based Mental Health Interventions and acceptance of AI-driven mental health interventions, *r*=–0.12 (N=466; *P*=.008). A significant but weak positive correlation was observed between the Effectiveness of AI in Mental Health Management and Stigma Associated With AI-Driven Mental Health Interventions, *r*=0.21 (N=466; *P*<.001). Similarly, a significant but weakly negative correlation was found between the Effectiveness of AI in Mental Health Management and Stigma Associated With Human-Based Mental Health Interventions, *r*=–0.20 (N=466; *P*<.001). Notably, there was a strongly significant relationship between the Effectiveness of AI in Mental Health Management and acceptance of AI-driven mental health interventions, *r*=0.55 (N=466; *P*<.001). A very weak but significant negative correlation was observed between Stigma Associated With AI-Driven Mental Health Interventions and Stigma Associated With Human-Based Mental Health Interventions, *r*=–0.11 (N=466; *P*=.01). A similarly weak but significant negative correlation was found between Stigma Associated With Human-Based Mental Health Interventions and acceptance of AI-driven mental health interventions, *r*=–0.09 (N=466; *P*=.04).

**Table 8:** Correlation analysis between each domain.

Domain		Knowledge and Awareness	Trust in AI-Based Interventions	Trust in Human-Based Interventions	Effectiveness of AI in Mental Health Management	Stigma Associated With AI-Driven Mental Health Interventions	Stigma Associated With Human-Based Mental Health Interventions	Acceptance of AI-Based Interventions
**Knowledge and Awareness About AI-Driven Mental Health Interventions**								
	*r*	1	0.348^a^	–0.005	0.194^a^	–0.009	–0.031	0.392^a^
	*P* value	—^b^	<.001	.91	<.001	.85	.50	<.001
**Trust in AI-Based Interventions**								
	*r*	0.348^a^	1	–0.129^a^	0.565^a^	0.079	–0.047	0.465^a^
	*P* value	<.001	—	.005	<.001	.09	.31	<.001
**Trust in Human-Based Interventions**								
	*r*	–0.005	–0.129^a^	1	–0.153^a^	0.052	–0.127^a^	–0.122^a^
	*P* value	.91	.005	—	.001	.26	.006	.008
**Effectiveness of AI in Mental Health Management**								
	*r*	0.194^a^	0.565^a^	–0.153^a^	1	0.208^a^	–0.200^a^	0.551^a^
	*P* value	<.001	<.001	.001	—	<.001	<.001	<.001
**Stigma Associated With AI-Driven Mental Health Interventions**								
	*r*	–0.009	0.079	0.052	0.208^a^	1	–0.114^c^	0.077
	*P* value	.85	.09	.26	<.001	—	.01	.01
**Stigma Associated With Human-Based Mental Health Interventions**								
	*r*	–0.031	–0.047	–0.127^a^	–0.200^a^	–0.114^c^	1	–0.094^c^
	*P* value	.50	.31	.006	<.001	.01	—	.04
**Acceptance of AI-Based Interventions**								
	*R*	0.392^a^	0.465^a^	–0.122^a^	0.551^a^	0.077	–0.094^c^	1
	*P* value	<.001	<.001	.008	<.001	.01	.04	—

^a^Correlation is significant at the .01 level (2-tailed).

^b^Not applicable.

^c^Correlation is significant at the .05 level (2-tailed).

### Opinions on the Purpose of Using AI in Mental Health

From Table 9, it is evident that a significant majority of participants preferred using AI in mental health for receiving general mental health tips (241/466, 51.7%), followed by its preference for initial assessment and screening (206/466, 44.2%). The least preferred use of AI-driven mental health interventions was for treatment, with only 24/466 participants (5.2%) expressing this opinion.

The benefits and concerns of AI-driven mental health interventions are listed in Textbox 1.

**Table 9:** Opinions on purposes for which the participants would use AI^a^ in mental health interventions (N=466).

Purpose	Values, n (%)
General mental health tips	241 (51.7)
Initial assessment and screening	206 (44.2)
Diagnosis	81 (17.4)
Follow-up	47 (10.1)
Treatment	24 (5.2)

^a^AI: artificial intelligence.

**Benefits and concerns of artificial intelligence–driven mental health interventions**

1. Benefits

- Get mental health tips
- Initial screening and early diagnosis
- Ease of accessibility
- Cost-effective and time-saving
- Personalized support
- Reduced stigma
- Reduces the burden on traditional mental health therapies
- Continuous monitoring and timely responses

2. Concerns

- Data privacy and security issues
- Lack of human touch and empathy
- Misdiagnosis
- Overdependence on technology

## Discussion

### Principal Findings

This study examined public perceptions of AI-driven mental health interventions, including initial assessment and testing, counseling, follow-up, disease diagnosis, treatment management, and the provision of general mental health tips, as well as their future prospects. A survey was conducted with 466 participants from India, aged 18-65 years. The data were analyzed based on gender and location, with the results compared and discussed accordingly.

The study revealed that AI-driven mental health interventions are still a relatively new concept in India, with many people still learning about their various aspects. The number of individuals who have accessed mental health interventions is lower compared with those who have not. Additionally, the public exhibits a lack of full trust in the information provided by these platforms, which may stem from limited knowledge or other factors, which are further explored in this study. Our results showed that, when comparing an AI-driven mental health platform with a human counselor, participants expressed greater comfort with a human counselor. Concerns regarding the privacy of their mental health information were more pronounced for the AI-driven platform than for a human counselor, with women showing more concern than men. Despite these privacy and human touch concerns, participants still believed that AI-driven interventions could play a role in reducing the burden on traditional mental health services. This suggests that while people in India are open to embracing AI-driven platforms, they are not yet ready to replace traditional human-based therapies with AI platforms. The majority of participants expressed a preference for AI-driven mental health interventions that would assist human counselors, rather than replace them. A detailed explanation of the findings by domain is provided in the following sections.

### Knowledge and Awareness About the Concept of AI-Driven Mental Health Interventions

According to a study conducted among medical students and doctors in India, a significantly larger proportion of female respondents reported feeling uninformed about the basics of AI technology and its applications in health care compared with their male counterparts [39]. However, this study contradicts these findings, revealing that the lack of awareness was higher among male participants than female respondents. It was also observed that people from rural, urban, and semiurban areas had low Knowledge and Awareness about various AI-driven mental health interventions. According to an article published by the Office for National Statistics (United Kingdom) [40], the percentage of adults who reported being able to identify when they are using AI ranged from 17% (1 in 6) to 21% in men, 31% in adults aged 16-29 years, and 22% in adults with a degree or equivalent qualification. As adults age, their awareness of AI usage decreases, with over half (55%) of those over 70 years reporting that they can rarely or never recognize when they are using AI [39]. This trend is consistent with our study, which found that people from various locations had low awareness of AI-driven mental health interventions. Additionally, a significant relationship was observed between awareness of AI-driven mental health interventions and age.

### Trust in AI-Driven Mental Health Intervention Versus Trust in Human-Based Mental Health Interventions

A study by Asan et al [41] highlighted that one of the major obstacles to the widespread use of AI in health care is the current lack of trust in these systems. Four factors influence Trust in AI-Driven Mental Health Interventions: ease of use, experience with AI, impact on jobs, and uncertainty about AI [42]. Our study found that there is moderate trust in both AI-driven mental health interventions and human-based interventions among people from rural, semiurban, and urban areas. There was a preference for moderate to low levels of Trust in AI-Driven Mental Health Interventions across both age groups, with individuals in the 18-26-year age group showing comparatively lower Trust in AI-Driven Mental Health Interventions than those in the older age group. By contrast, for human-based interventions, people in the 18-26-year age group demonstrated more trust than those in the 27-66-year age range. Additionally, men are less inclined than women to seek help for psychological issues [43,44]. According to Yang et al [44], gender has little impact on trust in medical AI technology. However, in our study, a significant relationship was found between gender and Trust in AI-Driven Mental Health Interventions, indicating that gender does influence Trust in AI-Driven Mental Health Interventions. It was also observed that the percentage of males with low Trust in AI-Driven Mental Health Interventions was significantly higher than that of females, suggesting that a greater proportion of females had a more trusting attitude toward AI-driven mental health interventions. By contrast, for human-based interventions, both males and females showed a moderate to high preference.

### Effectiveness of AI in Mental Health Management

According to a longitudinal study by Jain et al [45], it is important to recognize that, despite their ability to provide scalable, instantaneous support, chatbots are not a substitute for traditional treatment. Rather, they are intended to complement existing mental health services. In this study, a significant relationship was found between age and the perceived Effectiveness of AI in Mental Health Management. The data also revealed that, in the 18-26-year age group, fewer people considered AI-driven mental health interventions to be highly effective, compared with those who found them ineffective. However, in terms of gender, both males and females expressed mixed opinions, and no significant relationship was found. Across all 3 locations, participants reported a moderate level of effectiveness for AI-driven interventions.

### Stigma Toward AI-Driven Mental Health Services Versus Stigma Toward Human-Based Mental Health Interventions

A study on the stigma associated with mental health help–seeking behavior in the Indian population found that only 7.3% of young people in India report a mental disorder, and even fewer seek treatment for it [46]. In this study, no significant relationship was found between age and stigma toward AI-driven mental health interventions. However, a significant relationship was observed between age and stigma toward human-based mental health interventions, indicating that age influences the stigma associated with human-based interventions, but not with AI-based ones. When people disagree with medical practitioners, stigma can make it more difficult for them to seek treatment [19]. In the case of gender and stigma toward AI-driven mental health interventions, no significant relationship was found between the variables. However, the data show that the number of males who reported higher stigma was comparatively lower than those who reported less stigma toward AI-driven mental health interventions. Both males and females exhibited a similar pattern. However, in the case of human-based interventions, the number of people with higher stigma was comparatively greater than those with lower stigma. In terms of location, it was observed that the number of people with low stigma toward AI-driven mental health interventions was higher than those with higher stigma across all locations. Conversely, a majority of people in these locations reported a high stigma toward human-based interventions. AI in the mental health sector has the potential to address several challenges related to the availability, appeal, and accessibility of mental health treatments [21].

### Societal Acceptance and Likelihood of Future Use of AI

This domain addresses the feasibility of adopting AI-driven interventions in the near future. Behavioral intention, willingness, and usage of AI across various industries are substantially and positively influenced by perceived utility, performance expectation, attitudes, trust, and effort expectancy [47]. However, cultural variables must be considered when comparing acceptance studies conducted with different demographic groups [42,47-49]. The data from the study revealed that all age groups had a lower preference for AI-driven interventions. It also showed that people of all genders and from all locations expressed lower acceptance of AI in the near future.

### Relationship Between Various Domains

The study provided valuable insights into the relationship between various factors that affect attitudes toward AI-driven mental health interventions. A positive relationship was observed between Knowledge and Awareness About AI-Driven Mental Health Interventions and both Trust in AI-Driven Mental Health Interventions and acceptance of AI. Thus, a person’s overall awareness of and attitude toward the use of AI applications in medicine plays a significant role in determining their trust in and acceptance of AI-based interventions [42]. It was also found that Knowledge and Awareness About AI-Driven Mental Health Interventions positively influence perceptions of the effectiveness of AI-driven mental health interventions to some extent.

The results also indicated that Trust in AI-Driven Mental Health Interventions was negatively correlated with Trust in Human-Based Mental Health Interventions. This suggests that individuals who trust AI-driven interventions are less likely to have a trusting attitude toward human-based mental health interventions. However, it should be noted that there is a strong relationship between Trust in AI-Driven Mental Health Interventions and both their perceived effectiveness and acceptance. This indicates that if a person trusts AI-driven mental health interventions, they are likely to perceive these interventions as effective, with a higher likelihood of future usage [42,48,49].

The data also suggested that trust in human-based mental health interventions may negatively influence the perceived effectiveness of AI-driven mental health interventions, stigma associated with human-based interventions, and the acceptance of AI-driven interventions. This means that individuals with a high level of trust in human-based mental health interventions are less likely to view AI-driven interventions as effective, and their chances of accepting these AI-driven interventions would be lower [42]. Participants in similar studies have also reported that interacting with a chatbot would make them feel less open and honest compared with conversing with a human therapist [50]. This suggests that individuals with high trust in human-based mental health interventions are likely to experience lower stigma toward these interventions.

The results revealed a significant finding: the Effectiveness of AI in Mental Health Management is positively related to their acceptance. It was also observed that individuals with a high stigma toward human-based mental health interventions tend to have a lower stigma toward AI-based mental health interventions. Furthermore, the data showed that those with a high stigma toward human-based interventions are more likely to accept AI-driven mental health interventions.

### Preferences and Opinions of the Public for Using AI-Driven Mental Health

The open-ended responses to the survey were categorized, revealing that people mostly wanted to use AI-driven mental health interventions for receiving mental health tips and for initial assessment and screening. This further supports the earlier finding that the population views AI-driven interventions as tools to assist human mental health professionals, helping to reduce their workload rather than replacing traditional therapies entirely [51]. Our study suggests that one reason the general public prefers AI over human counselors is its accessibility: AI is available anytime and can be accessed more easily [32,52]. It is also seen as more affordable than human counseling and time-saving. Another important factor is that people often prefer a nonjudgmental approach in their counselor, which they believe AI can provide more effectively than humans [53,54]. Additionally, as previously mentioned, consulting an AI involves less stigma, as fewer people are involved in the process [55]. AI can also offer continuous monitoring, which is not possible with human counselors. This makes follow-up an essential feature that the general public values. Textbox 2 presents a few excerpts from participants regarding what they find beneficial in an AI-driven mental health platform.

Furthermore, one of the biggest concerns people have about using AI for mental health is related to data privacy and security [56], as well as the lack of human touch and empathy (Textbox 3).

**Excerpts from participants regarding the benefits of artificial intelligence.**

- When the patient knows that no human is there to judge them, will decrease social desirability related concerns.
- AI won’t get overwhelmed with emotions, if the language model is trained effectively, it can produce greatly accurate results with almost no biases.
- Very objective analysis, won't get implicated in the client’s emotions, solves the problem of compassion fatigue, can also be used to help therapists.
- Therapists can supplement AI with their current practices to make it more efficient.
- Sometimes a face-to-face interaction on such matters can be a little awkward. But such things might become easier in an AI-based environment.

**Participants’ concerns regarding using artificial intelligence.**

- Humans tend to understand the impact of emotions more than the AI since we’re the ones undergoing it.
- Emotions are not programmed and can be different for each individual and situations. I don’t think AI can capture the depth of it.
- Sometimes face/gestures speak more than words. But such things can’t be captured by AI.
- AI tools are only as effective as the data that they are trained on. Therefore, if the data is biased, the results will also be biased, leading to incorrect diagnosis and treatment recommendations.

These transcripts highlight the importance of human emotions and face-to-face interactions in mental health care. An important ethical concern is preserving the human aspect of treatment while using AI as a tool. The therapeutic alliance between patients and therapists should be strengthened by AI, not replaced [57,58]. Additionally, there is a fear of misdiagnosis, and many people are concerned about the heavy reliance on technology in such interventions.

### Limitations and Future Directions

The scope of this study is limited to India, and the sample was recruited through social media platforms, excluding individuals who are not active on social media. Additionally, the sample did not have an equal representation of urban and rural populations. Future studies could address these limitations by using random sampling methods and targeting a broader segment of the population. The concept of AI-driven mental health interventions is still in its formative phase, so public perceptions are based on the current state of these interventions and their hypothetical future prospects. These views are relevant only in the present context. The efficacy of ongoing mental health interventions, such as support provided through app-based chatbots, could provide more empirical evidence about their real-world impact.

### Conclusions

There is an overall lack of awareness regarding mental health interventions among the public. According to public opinion, AI could be a viable option for prevention, primary assessment, and ongoing mental health maintenance until more advanced versions are tested for counseling and therapy. However, people tend to place more trust in traditional, human-based mental health professionals. While stigma is a barrier to seeking help from human mental health professionals, it is less of an issue when accessing AI-driven interventions. However, emotions, feelings, and empathy are essential aspects of mental health care that AI has not yet been able to replace. A model integrating AI with human mental health professionals would be a more compelling approach. AI is perceived to have a positive impact on accessibility, availability, and destigmatization. Knowledge and trustworthiness are key factors in increasing the acceptance and effectiveness of AI in mental health management.

## Data Availability

The data sets generated or analyzed during this study are available from the corresponding author upon reasonable request.

## Abbreviations

AYUSH: Ayurveda, Yoga, Naturopathy, Unani, Siddha, and Homeopathy
LGBTQ: lesbian, gay, bisexual, transgender, and queer or questioning
NIMHANS: National Institute of Mental Health and Neurosciences
NTMHP: National Tele-Mental Health Program
